# Determination of 17 Organophosphate Pesticide Residues in Mango by Modified QuEChERS Extraction Method Using GC-NPD/GC-MS and Hazard Index Estimation in Lucknow, India

**DOI:** 10.1371/journal.pone.0096493

**Published:** 2014-05-08

**Authors:** Ashutosh K. Srivastava, Satyajeet Rai, M. K. Srivastava, M. Lohani, M. K. R. Mudiam, L. P. Srivastava

**Affiliations:** 1 Pesticide Toxicology Laboratory, CSIR-Indian Institute of Toxicology Research, (Council of Scientific and Industrial Research, Govt. of India), Mahatma Gandhi Marg, Lucknow, India; 2 Department of Biotechnology, Integral University, Lucknow, India; Indian Institute of Toxicology Research, India

## Abstract

A total of 162 samples of different varieties of mango: Deshehari, Langra, Safeda in three growing stages (Pre-mature, Unripe and Ripe) were collected from Lucknow, India, and analyzed for the presence of seventeen organophosphate pesticide residues. The QuEChERS (Quick, Easy, Cheap, Effective, Rugged and Safe) method of extraction coupled with gas chromatography was validated for pesticides and qualitatively confirmed by gas chromatography- mass spectrometry. The method was validated with different concentrations of mixture of seventeen organophosphate pesticides (0.05, 0.10, 0.50 mg kg^−1^) in mango. The average recovery varied from 70.20% to 95.25% with less than 10% relative standard deviation. The limit of quantification of different pesticides ranged from 0.007 to 0.033 mg kg^−1^. Out of seventeen organophosphate pesticides only malathion and chlorpyriphos were detected. Approximately 20% of the mango samples have shown the presence of these two pesticides. The malathion residues ranged from ND-1.407 mg kg^−1^ and chlorpyriphos ND-0.313 mg kg^−1^ which is well below the maximum residues limit (PFA-1954). In three varieties of mango at different stages from unpeeled to peeled sample reduction of malathion and chlorpyriphos ranged from 35.48%–100% and 46.66%–100% respectively. The estimated daily intake of malathion ranged from 0.032 to 0.121 µg kg^−1^ and chlorpyriphos ranged from zero to 0.022 µg kg^−1^ body weight from three different stages of mango. The hazard indices ranged from 0.0015 to 0.0060 for malathion and zero to 0.0022 for chlorpyriphos. It is therefore indicated that seasonal consumption of these three varieties of mango may not pose any health hazards for the population of Lucknow, city, India because the hazard indices for malathion and chlorpyriphos residues were below to one.

## Introduction

Mango (*Mangifera indica*) is one of the most common and highly consumable tropical fruits of India. It is rich in carotenoid, minerals, carbohydrates and vitamins. India ranked first in mango production in the world during 2010-11 [1]. Lucknow, the capital of Uttar Pradesh, India, is the largest producer of mangoes producing around 3469.5 metric tons, the productivity being about 12.8 tones/hectare [2]. There are many insect pest pressures for mangos grown in this region of India requiring the use of pesticides to increase the productivity [3]. Therefore, for obtaining good quality and high productivity of mango fruits, the commercial cultivation of mango receive frequent application of various contact and systemic pesticides throughout the cropping season [4]. Most pesticide residues find their way into the human body through fruits, vegetables, cereals, water and other food commodities. Thus, analysis of pesticide residues in food commodities and other environmental samples have become an essential requirement for consumers, producers and food quality control authorities [5]. Due to increased use of pesticide in the orchard, pesticide residues may remain in the raw fruits and their products such as juices, nectar, jellies and ice cream ‘pose to be poisonous hazards to human health owing to their toxicity’ [6]–[7]. To increase foreign trade under WTO regime, it is imperative to produce pesticide free mango [8]. Among the various pesticides, organophosphates (OPs), are the most extensively used insecticides in many crops including mango. Due to low persistence and high bio-efficiency of organophosphates, many farmers regularly use this group of pesticides for various vegetables and fruit crops. The continuous use of pesticides has caused deleterious effects the ecosystem [9]. Because of wide spread use of pesticides, the presence of their toxic residues have been reported in various environmental component/commodities [5], [10]–[11]. Public awareness of health hazards posed by pesticide residues in fruits and vegetables has led to the development of many analytical methods. [5], [7], [8], [12]–[16]. Method validation is an important requirement in chemical analysis. The analyst must generate information to show that a method intended for this purpose is capable of providing adequate specificity, accuracy and precision at relevant analyte concentrations in appropriate matrices. In the present study, an attempt has been made to validate modified QuEChERS method using ethyl acetate (EtOAc) for the extraction. In the method QuEChERS is reported that the acetonitrile is not compatible with system of gas chromatography due to high volume spray, which significantly increases the internal pressure of the chromatographic system. So we adapt the QuEChERS method employing the ethyl acetate solvent.

**Figure 1:**
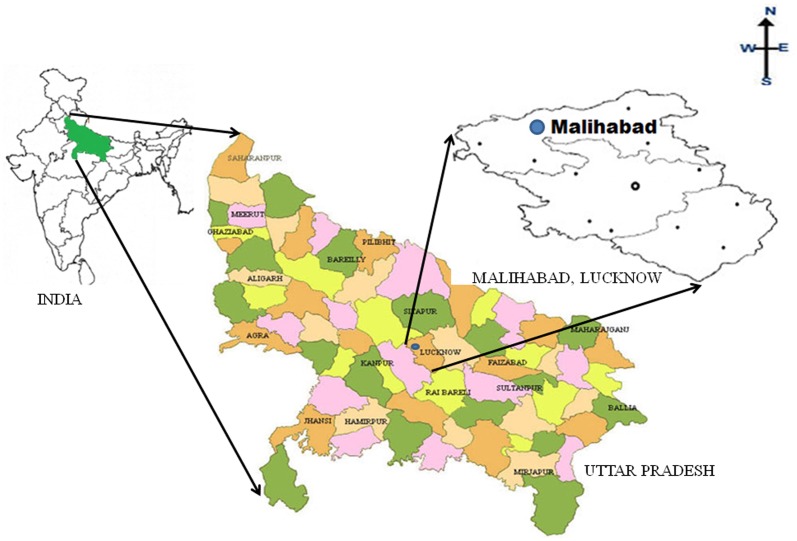
Map showing sampling site Malihabad, Lucknow, Uttar Pradesh, India.

Seventeen organophosphate pesticides like dichlorvos, phorate, phorate-sulfone, phorate-sulfoxid, dimethoate, diazinon, methyl-parathion, chlorpyrifos-methyl, fenitrothion, malathion, chlorpyrifos, chlorfenvinfos, profenofos, ethion, edifenophos, anilophos and phosalone in mango fruits were analysed by Gas chromatography using Nitrogen Phosphorus Detector (GC-NPD). Majority of these pesticides are being used in mango orchards during spraying [17]–[18]. The validated method has been applied to determined 17 OPs residues in three delicious varieties of mango like Deshehari, Langra and Safeda of Malihabad, Lucknow, Uttar Pradesh, India as these varieties are prone to insect pests and consumed largely (Fig. 1), determining estimated daily intake (EDI) and hazard index of detected OPs residues for the consumption of mangoes by local population of Lucknow, India.

## Materials and Methods

### Ethics Statement

No specific permissions were required for these locations/activities. There is no requirement for ethical permission for this study.

### Chemicals and Pesticide Standards

All solvents like n-hexane, acetone and ethyl acetate (HPLC grade) were purchased from Sigma-Aldrich. Co. USA, Spectrochem Pvt. Ltd. India and were glass distilled before use. Acetone was refluxed over potassium permanganate for 4 hr and then distilled. Sodium chloride (NaCl), anhydrous sodium sulphate (Na_2_SO_4_) and magnesium sulphate (MgSO_4_) were procured from Himedia Pvt. Ltd. India. Before use sodium sulphate (Na_2_SO_4_) and magnesium sulphate (MgSO_4_) were purified with acetone and baked for 4 hr @ 600°C in muffle Furness to remove possible phthalate impurities. Primary secondary amine (PSA) bondasil 40 µm part 12213024 of Varian was used.

Pesticide standards (Dichlorvos 98.9%, phorate 96.0%, phorate-sulfone 96.8%, phorate-sulfoxid 94.8%, Dimethoate 99.6%, diazinon, methyl-parathion 99.7%, chlorpyrifos-methyl 99.9%, fenitrothion 95.2%, Malathion 97.2%, chlorpyriphos 99.9%, chlorofenvinfos 98.7%, profenofos 95.0%, Ethion 97.8%, Edifenphos 99.9%, anilophos 97.5% and phosalone 95.2%) were purchased from Supelco Sigma Aldrich USA, Fluka Sigma-Aldrich Schweis and Rankem Pvt. Ltd. New Delhi, India.

### Sample collection

A total of 162 mango samples like Deshehari, Langra and Safeda of pre mature, unripe and ripe, from three different orchards of Malihabad, Lucknow, Uttar Pradesh, India were collected. (Fig. 1). Three batches of pre-mature, unripe and ripe mangoes (6 samples per batch) of individual varieties were taken for analysis. Samples of Mangoes (6 samples x 3 conditions/batches x 3 varieties x 3 orchards = 162 samples) were brought to the laboratory and analyzed as soon as possible or stored in refrigerator at 4±2°C until analysis.

### QuEChERS Sample Preparations

**Figure 2:**
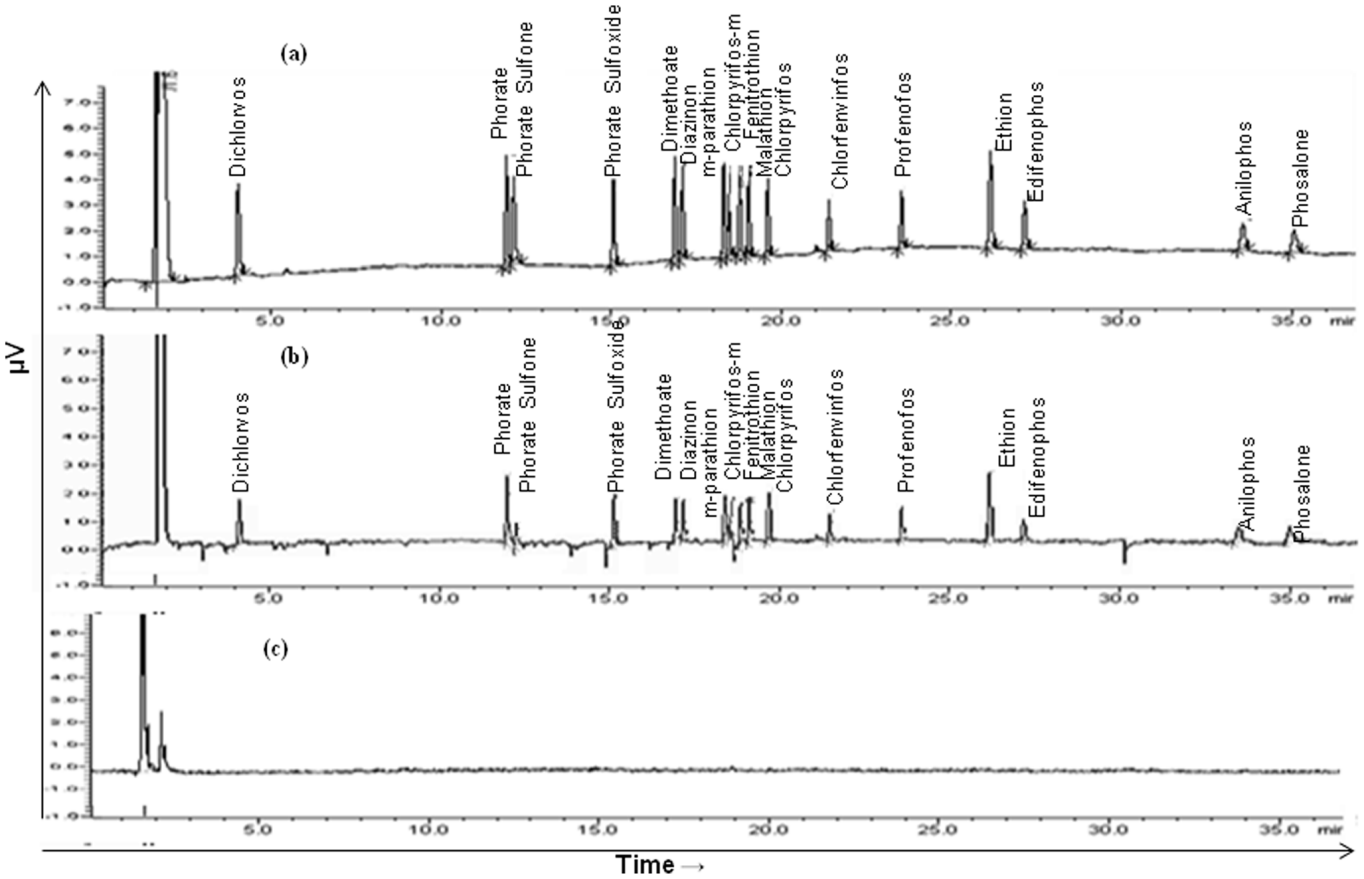
GC-NPD Chromatogram of (a) standard solution of 17 pesticides (0.5 mg l^−1^). (b) spiked mango sample (0.10 mg kg^−1^) and (c) blank mango sample.

The unpeeled and peeled mango samples (50 g) of each variety were chopped and grinded in Warring blander. Macerated sample of each mango in triplicates were extracted and cleaned by QuEChERS method as follows: 10±0.1 g macerated sample was mixed with 10 ml ethyl acetate, 4 g activated anhydrous MgSO_4_, 1.0 g activated NaCl in centrifuge tube and shaken for 10 min. at 50 rpm on rotospin. The extract was centrifuged for 10 min at 8,000 rpm. 1 ml supernatant of extract was cleaned with the mixture of 50 mg PSA, 150 mg anhydrous MgSO_4_ and 10 mg activated charcoal (application of activated charcoal in the stage of clean up to remove pigments from matrix). The extract was again shaken for 10 min at 50 rpm on rotospin and centrifuged for 10 min at 8,000 rpm. The supernatant was collected in GC vial for the analysis. Steps of extraction and clean up using a longer agitation related to the use of ethyl acetate solvent, which is less efficient than the acetonitrile with regard to power extraction of pesticides in the partitioning process is documented [12]. The use of larger time in the agitation may have been an attempt to maximize the extraction of pesticides, but promoted a significant increase in analysis time compared with the original method. 1 µl clean extract was injected in gas chromatography equipped with–nitrogen phosphorus detector (NPD) for the analysis OP pesticide residues. (Fig. 2)

### GC-NPD Analysis

Residues were analyzed on Shimadzu GC-2010 equipped with fused silica capillary column, DB-1 (30 mt.×0.25 mm. id) coated with 100% dimethylpolysiloxane (0.25 µm film thickness) using NPD. General operating conditions were as follows; Injector port temperature: 250°C; detector temperature 280°C; carrier gas Nitrogen (N_2_); flow 1.46 ml min^−1^; Hydrogen (H_2_) makeup is 30 ml min^−1^ and zero air 60 ml min^−1^, (zero air has less than 0.1 ppm hydrocarbons, to decreases the background noise level and gives the baseline much better stability, considerably increasing detector sensitivity and ensuring precise analytical results) column temperature program: initially 130°C hold 2 min, increase at 5°C/min to 170°C hold 3 min, then increase 220°C min^−1^ at 5°C min^−1^, hold for 14 min; injection volume: 1 µl split ratio 1∶5. The total run time was 37 min and Shimadzu, GC Solution software was used for instrument control and data analysis. Quantification of the pesticides was done by peak area using the standard method.

### GC-MS Confirmation

A Perkin Elmer GC-MS consisting of Auto system XL Gas Chromatograph with a Turbo Mass Spectrometer was used for analysis. The column used in this study is Elite-5MS fused-silica capillary column (30 m×0.32 mm I.D., 0.25 mm film thickness). Carrier gas used was helium (purity 99.999%) with a flow rate of 1.6 ml min^−1^. A 1 µl aliquot of the extract was injected using the splitless mode. The oven temperature program is 100°C for 1 min and then @ 20°C min^−1^ to 210°C and hold for 1 min; then @45°C min^−1^ to 300°C and hold for 1 min. The total runtime of the GC is 9.5 min. Base peaks 173, 158, 127 m/z for malathion as target ion as well as 314, 286, 258 m/z for chlorpyriphos were noticed as qualifiers in selective ion mode (SIM) for analysis. The injector temperature was set at 300°C. The transfer line and source temperature was set at 280°C and 230°C respectively. Solvent delay for MS is 5 min.

### Method validation

The validation of the analytical method was performed by the accuracy, precision, linearity and limit of detection (LOD), quantification (LOQ). All the analysis was carried out using the same blank samples of mango fruit.

Accuracy and precision data were obtained with recovery studies by spiking samples with pesticide standards at levels of 0.05, 0.1, and 0.5 mg kg^−1^. The spiked and control samples were analyzed in five replicates. Precision of the method was evaluated through the relative standard deviations (%RSD) associated with pesticides measurements during recovery.

Linearity was determined by plotting calibration curve with standard solutions in n-hexane containing five different concentrations (0.025, 0.05, 0.1, 0.5 and 1.0 mg L^−1^). Five injections were made at each of the five concentration levels.

The limits of detection (LOD) and Quantification (LOQ) were calculated according standard guidelines [19]–[20]. Five independent analysis of mango samples spiked with mixture of 17 OPs at the level of 0.05, 0.1, and 0.5 mg kg^−1^ were performed for the percentage recovery of each pesticides. The LOD and LOQ were calculated at the spiking level of 0.1 mg kg^−1^ from the standard deviation of this determination. Table 1

**Table 1:** Fortification experiment of organophosphate pesticides residues from spiked mango at different levels (recovery and repeatability), limit of detection (LOD), limit of quantification (LOQ), maximum residues limits (MRL) and acceptable daily intake (ADI).

P Pesticides	Fortification level (mg kg^−1^)	Recovered	(%) Recovery (n = 3)	LOD (mg kg^−1^)	LOQ (mg kg^−1^)	%RSD^a^	MRL^b^ (mg kg^−1^)	ADI^c^ (mg kg^−1^day^−1^)
Dichlorvos	0.05	0.039±3.22	78.90					
	0.10	0.080±3.22	80.23	0.003	0.010	4.42	0.1	0.004
	0.50	0.423±3.22	84.57					
Phorate	0.05	0.036±3.22	72.33					
	0.10	0.075±3.22	75.45	0.002	0.007	3.17	0.05	0.0005
	0.50	0.391±3.22	78.20					
Phorate Sulfone	0.05	0.035±3.22	70.22					
	0.10	0.071±3.22	71.45	0.010	0.033	3.84	0.05	0.0005
	0.50	0.373±3.22	74.60					
Phorate Sulfoxide	0.05	0.040±3.22	80.35					
	0.10	0.080±3.22	80.00	0.003	0.010	5.35	0.05	0.0005
	0.50	0.426±3.22	85.25					
Dimethoate	0.05	0.039±3.22	78.23					
	0.10	0.080±3.22	80.45	0.003	0.010	6.95	2.0	0.002
	0.50	0.425±3.22	85.10					
Diazinon	0.05	0.043±3.22	85.28					
	0.10	0.086±3.22	86.30	0.001	0.004	9.50	NA	0.02
	0.50	0.450±3.22	90.00					
m-Parathion	0.05	0.045±3.22	91.11					
	0.10	0.088±3.22	88.35	0.001	0.004	3.23	0.2	0.003
	0.50	0.467±3.22	93.45					
Chlorpyrifos-m	0.05	0.043±3.22	85.25					
	0.10	0.084±3.22	83.70	0.003	0.010	4.91	NA	0.01
	0.50	0.444±3.22	88.90					
Fenitrothion	0.05	0.036±3.22	72.90					
	0.10	0.070±3.22	70.20	0.010	0.033	5.37	0.5	0.005
	0.50	0.372±3.22	74.50					
Malathion	0.05	0.041±3.22	81.25					
	0.10	0.082±3.22	82.50	0.002	0.008	8.49	4.0	0.02
	0.50	0.428±3.22	85.55					
Chlorpyrifos	0.05	0.045±3.22	90.57					
	0.10	0.092±3.22	92.40	0.001	0.004	6.70	0.5	0.01
	0.50	0.476±3.22	95.25					
Chlorfenvinfos	0.05	0.044±3.22	88.10					
	0.10	0.091±3.22	90.75	0.003	0.010	6.48	NA	NA
	0.50	0.466±3.22	93.25					
Profenofos	0.05	0.042±3.22	83.25					
	0.10	0.080±3.22	80.25	0.004	0.013	8.68	NA	NA
	0.50	0.417±3.22	83.45					
Ethion	0.05	0.040±3.22	80.25					
	0.10	0.080±3.22	80.40	0.005	0.017	8.23	2.0	NA
	0.50	0.428±3.22	85.70					
Edifenophos	0.05	0.046±3.22	92.34					
	0.10	0.090±3.22	90.40	0.001	0.005	7.23	NA	NA
	0.50	0.467±3.22	93.50					
Anilophos	0.05	0.039±3.22	77.72					
	0.10	0.079±3.22	79.45	0.002	0.007	8.18	NA	NA
	0.50	0.416±3.22	83.20					
Phosalone	0.05	0.042±3.22	84.22					
	0.10	0.080±3.22	80.50	0.003	0.010	9.61	5.0	0.02
	0.50	0.431±3.22	86.25					

a RSD-Relative Standard Deviation.

b PFA - Prevention of Food Adulteration Act Govt. of India, 1954 [31].

c [32].

NA- Not Available.

In order to maintain analytical quality control for each sample batch a spiked sample (similarly used in the recovery study) was analyzed simultaneously. Batch results were considered unsatisfactory when the sample used as quality control had low recovery.

### Hazard index estimation

Health risk estimations were performed on the basis of pesticide analysis data obtained in present study and annual fruit intake per person. The estimated daily intake (EDI) of pesticides is the toxicological criteria for their exposure. It is calculated as per international guidelines [21]–[23] using the equation: EDI =  C x F/D x W where C is the mean of individual pesticide concentration (mg kg^−1^); F is mean annual intake of fruit per person (kg); D is days in a year (365 days) and W is the mean human body weight (60 kg). The annual intake as the total fruits per person is 9.5 kg per year according to Indian survey perform in years 2005–2006 [24]–[26]. The EDI (mg kg^−1^ day^−1^) as obtained were used to estimate the hazards index by dividing them to their corresponding value of known acceptable daily intake (Hazard index  =  EDI/ADI).

### Statistical Analysis

The data were statically analyzed by using one way ANOVA. Criterion of significance was taken as P<0.05, P<0.01 and P<0.001. All statistical calculations have been done using IBM SPSS statistics version 20.

## Results and Discussion

### Validation of QuEChERS method

Fig. 2 (a.b,c) showing the representative chromatogram for standard of OP mixture, spiked and blank mango fruit samples. Adequate separation of the 17 OPs was achieved. No interference peaks were obtained in the blank sample chromatogram at the same retention time of the target compounds.

All OPs showed linearity ranged (0.025 to 1.0 mg kg^−1^) with the coefficient co-relation more than 0.997 (r). The relative standard deviation (RSD) of the three replicate injections ranged from 3.17 to 9.50% showing good repeatability.

Table 1, shows that recovery data and repeatability of 17 OPs analyzed at three different spiking levels. The recovery ranged from 70.20 to 93.50%. The overall recovery was more than 70% (except phorate sulfone and fenitrothion) at lower spiking level 0.05 mg kg^−1^ with RSD below 10% which represent satisfactory repeatability of the method for all pesticides (Barakat et al. 2007). It is observed that LOD and LOQ for 17 pesticides ranged from 0.001 to 0.010 mg kg^−1^ and 0.007 to 0.033 mg kg^−1^ respectively. It is interesting to note that, LOQ analyzed of all 17 OPs was lower than their respective MRL's established by PFA 1954, Govt. of India (Table 1). Few studies have been reported for the presence of organophosphate residues in mango using various extraction technique used in gas chromatographic analysis [13], [20], [27]–[28].

In the present study, the QuEChERS sample preparation and GC analysis using ethyl acetate solvent has been validated. The results indicate that the QuEChERS sample preparation method coupled with GC-NPD analysis is suitable for the determination of 17 OPs viz; dichlorvos, phorate, phorate-sulfone, phorate-sulfoxide, dimethoate, diazinon, methyl-parathion, chlorpyrifos-methyl, fenitrothion, malathion, chlorpyrifos, chlorofenvinfos, profenofos, ethion, edifenophos, anilophos and phosalone. Successful use of QuEChERS method has been reported for the analysis of pesticide residues in high sugar content matrices like honey and sugarcane juice [27], [29]. Similarly the present study revealed good result by applying QuEChERS method for the analysis of various organophosphate in ripe mango fruit which are enriched in high sugar content.

### Pesticide residue in various stages of the mango

Pesticides are commonly sprayed to mango trees at pre-mature, unripe and one month before the harvest. Therefore, mangoes of all three stages were studied. Analysis was done in samples of three different varieties of mangos (deshehari, langra and safeda) in triplicates for the presence of pesticides residues and values are given in Table 2. Out of seventeen analyzed pesticide only two pesticides malathion and chlorpyrifos were detected in three varieties of mangoes.

**Table 2:** Level of malathion and chlorpyrifos residues (mg kg^−1^) in different species of mango fruits and their percent reduction after peeling.

Mango	Pre mature n = 6		Unripe n = 6		Ripe n = 6		Samples A
	Malathion Mean ± SD	Chlorpyrifos Mean ± SD	Malathion Mean ± SD	Chlorpyrifos Mean ± SD	Malathion Mean ± SD	Chlorpyrifos Mean ± SD	Detected/Analysed
**Deshehari**							**14/54**
Unpeeled	0.969±0.050{3}	0.090±0.035{4}	0.062±0.020{3}***	0.028±0.005{2}**	0.021±0.010{2}***	ND	
	(ND-1.185)	(ND-0.223)	(ND-0.197)	(ND-0.113)	(ND-0.304)		
Peeled	0.011±0.005{3}^###^	0.048±0.050{4}	0.040±0.025{3}*	0.014±0.025{2}	ND	ND	
	(ND-0.046)	(ND-0.142)	(ND-0.128)	(ND-0.057)			
% reduction	98.86	46.66	35.48	50.00	100	ND	
**Langra**							**11/54**
Unpeeled	0.049±0.030{2}	0.160±0.070{2}	0.679±0.125{5}***	ND	0.213±0.050{2}***	ND	
	(ND-0.294)	(ND-0.313)	(ND-1.407)		(ND-0.853)		
Peeled	ND	ND	0.272±0.015{5}^###^	ND	ND	ND	
			(ND-0.932)				
% reduction	100	100	59.94	ND	100	ND	
**Safeda**							**7/54**
Unpeeled	ND	ND	0.480±0.015{5}	ND	0.147±0.080{2}	ND	
			(ND-0.908)		(ND-0.591)		
Peeled	ND	ND	0.139±0.025{5}^###^	ND	0.035±0.650{2}	ND	
			(ND-0.239)		(0.105)		
% reduction	ND	ND	71.04	ND	76.19	ND	
**Total Samples Detected/Analysed**							**32/162**

A Total samples of each variety of mango.

Values within small parentheses ( ) indicates range of residues and middle {} indicates number of samples detected with pesticides.

* p<0.05, ** p<0.01, *** p<0.001, ^###^p<0.001.

### Deshehari

#### Comparison between peeled and unpeeled samples

The results of Table 2 showed the presence of malathion and chlorpyrifos residues in different stages of mango. The mean concentration of malathion and chlorpyrifos was 0.969 (ND-1.185) and 0.090 (ND-0.223) mg kg^−1^ respectively in pre-mature unpeeled samples. However, malathion and chlorpyrifos residues were 0.011(ND-0.046) and 0.048 (ND-0.142) mg kg^−1^ in the pre-mature peeled mango. It is observed that there was a reduction of 98.86% malathion and 46.66% chlorpyrifos after the peeling of samples. The level of malathion residue in unpeeled to peeled mango samples was significantly (P<0.001) reduced and no significant variation was observed for chlorpyrifos. However in unripe unpeeled mangoes the mean concentration of malathion was 0.062 (ND-0.197) mg kg^−1^ and chlorpyrifos was 0.028 (ND-0.113) mg kg^−1^. The residues of malathion and chlorpyrifos were 0.040(ND-0.128) mg kg^−1^ and 0.014 (ND-0.057) mg kg^−1^ in unripe peeled mangoes. The percent reduction of malathion was 35.48 and chlorpyrifos was 50.00 through peeling in terms of mean values. The variation in level of malathion and chlorpyrifos was not significant. In ripe unpeeled mangoes, the residue of malathion 0.021 mg kg^−1^ (ND-0.304) was observed while chlorpyrifos was below to detectable level. None of these two pesticides were detected after the peeling of ripe mangoes. It is interesting to note that in ripe mangoes, the OP residues were least as compared to unripe or pre-mature mangoes. It may be because of judicious use of pesticides in the latent harvesting period of mangoes.

#### Comparison between pre-mature, unripe and ripe mango samples

The variation of malathion residues in unpeeled unripe and ripe samples was significant (P<0.001) in comparison to pre-maturate samples. Similarly, residues of chlorpyrifos was also significant (P<0.05) in unripe Deshehari mango. Whereas only malathion was significant (P<0.01) in unripe peeled mango samples.

### Langra

#### Comparison between peeled and unpeeled samples

The mean concentration of malathion and chlorpyrifos was 0.049 (ND-0.294) mg kg^−1^ and 0.160 (ND-0.313) mg kg^−1^, respectively in pre-mature unpeeled langra mango samples. The cent percent malathion and chlorpyrifos residues were reduced from unpeeled to peeled samples. However in unripe unpeeled mango the mean concentration of malathion was 0.679 (ND-1.407) mg kg^−1^ and 0.272 (ND-0.932) mg kg^−1^ in peeled mango samples. The percent reduction of malathion was 59.94 through peeling with respect to their mean values. The reduction in the level of malathion was significant (P<0.001) in unpeeled to peeled mango samples. Chlorpyrifos was not detected in unripe, unpeeled and peeled mango samples. In ripe mango malathion was detected 0.213 (ND-0.853) mg kg^−1^ in unpeeled sample with 100% reduction from unpeeled to peeled samples. However, chlorpyrifos was again not detected in ripe unpeeled and peeled samples.

#### Comparison between pre-mature, unripe and ripe mango samples

The variation of malathion residues in unpeeled samples of pre-maturate, unripe and ripe mangoes was significant (P<0.001), if compared with premature Langra mango samples.

### Safeda

#### Comparison between peeled and unpeeled samples

Malathion and chlorpyrifos residues were detected in unripe and ripe stages of mangoes. In unripe unpeeled mango malathion residue was 0.480(ND-0.908 mg kg^−1^) and in peeled samples the residue was 0.139(ND-0.239 mg kg^−1^) with 71.04% reduction of their mean values after peeling of the samples. The reduction of malathion was significant (P<0.001) in unpeeled to peeled mango samples. In ripe mangoes, malathion was 0.147(ND-0.591 mg kg^−1^) in unpeeled samples and 0.035(ND-0.105 mg kg^−1^) in peeled samples with 76.19% reduction is seen in their mean values after peeling. Malathion and chlorpyrifos residues were not detected in unpeeled and peeled pre-mature mangoes. However, chlorpyrifos was below detection limit in all three stages of safeda mango.

#### Comparison between pre-mature, unripe and ripe mango samples

The malathion and chlorpyrifos residues in unpeeled and peeled pre-maturate, unripe and ripe mangoes was not significant in safeda mango samples.

The texture, sugar and water content of mango may vary on the maturity, varieties and cultivars of the fruits. The texture and water content play an important role in trapping of pesticides and recovery efficiency.

**Figure 3:**
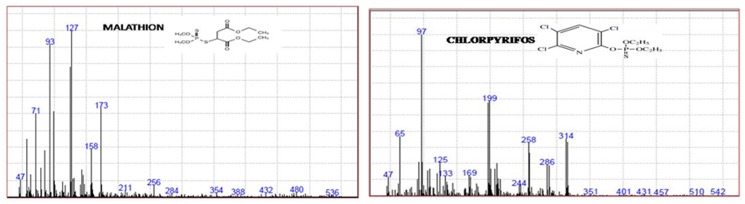
GC-MS chromatogram showing confirmation of malathion and chlorpyrifos.

The results of the method validation indicated that the QuEChERS sample preparation coupled with the GC-NPD analysis is suitable for the determination of 17 OPs in mango samples. In the present study none of pesticide residues accept malathion and chlorpyriphos were detected in 162 mango samples. Therefore, malathion and chlorpyrifos were further confirmed on GC-MS (Fig. 3).

Table 3 shows average estimated daily intake and hazard index for malathion and chlorpyrifos in pre-mature, unripe and ripe mango fruits. Hazard indices were calculated 0.0037, 0.0060 and 0.0015 for malathion and 0.0022, 0.0003 and zero for chlorpyrifos respectively in pre-mature, unripe and ripe mangoes. It is therefore indicated that the consumption of mango may not pose health hazards for the population of Lucknow city, India as hazard indices for malathion and chlorpyrifos in all three stages of mangoes were below one [30]. The total concentration of malathion and chlorpyrifos residues were 0.520 and 0.057 mg kg^−1^ which are below to maximum residues limits, 4.0 mg kg^−1^ and 0.5 mg kg^−1^ [31]. It is further stated that there is no health risk associated with malathion and chlorpyrifos residues after the consumption of mango fruits.

**Table 3:** Estimated daily intake and hazard index of malathion and chlorpyriphos residues in mango.

Mango	Average Pesticide concentration in three varieties of Mangos (ΣC) (µg kg^−1^)		Mean annual intake of fruit a ^,^ b per-person/day (kg) (F)	No. of Days in year (D)	Average weight of person (W)	ADI (µg kg^−1^ bw daily)		EDI (µg kg^−1^ bw daily)		Hazard index		Hazard Risk	
	Mala	CPF				Mala	CPF	Mala	CPF	Mala	CPF	Mala	CPF
Pre-Mature	172	50	9.5	365	60	20	10	0.075	0.022	0.0037	0.0022	No	No
Unripe	279	7	9.5	365	60	20	10	0.121	0.003	0.0060	0.0003	No	No
Ripe	69	0	9.5	365	60	20	10	0.030	0.00	0.0015	0.00	No	No

Mala  =  Malathion; CPF  =  Chlorpyriphos.

a From National Nutrition Monitoring Board (2008).

b National Sample Survey Organization (2000).

## Conclusion

The validated QuEChERS method applied in the present study fulfils the established criteria for sensitivity and confident identification of organophosphorus pesticides at low level in matrix with high sugar content like mango. The results revealed that none of OPs pesticides except traces of malathion and chlorpyrifos residues were present in mango fruits. It is also observe that peeling has great influence in reduction of pesticide residues in the pulp. To avoid adverse effects on public health it is necessary to set up control measures so as to make sure that each pesticide residues should be below MRL in the fruits to be marketed. Therefore the study has explored the significant information regarding the analysis of 17 OPs residues in different varieties of mango fruits of Malihabad, Lucknow region of India. It is further observed that mango fruits appear to be safe from OP pesticides residues as revealed by EDI and hazard index. In this manner one can assume that there is no apparent risk to the health of consumers of mango, as two detected pesticides have hazard index below to one. It is therefore, suggested that regular evaluation of pesticide residues should be carried out on mango fruits at national level for the planning of future policies about the use of pesticides in mango orchards and enables pesticide free fruits.
